# Supplementary material for: Ethical compliance and institutional policy support for artificial intelligence integration in African TVET Education: A structural equation modeling approach
Source: PLoS One. 2025 Dec 19;20(12):e0335747. doi: 10.1371/journal.pone.0335747 (PMC12716723; doi:10.1371/journal.pone.0335747)
Supplement: Attachment — Submitted filename: Review.docx [file pone.0335747.s001.docx]

Thanks for submitting your article to PLOS ONE journal. The topic titled **“Ethical Compliance and Institutional Policy Support for Artificial Intelligence Integration in African TVET Education: A Structural Equation Modeling Approach”** this topic is timely and contribute to the body of knowledge. The introduction and literature review was well articulated with robust analysis and good discussion section. Below are some observations to strengthen the draft to meet international readership

**Introduction section**

1. Break up long paragraphs for better readability and academic flow.
2. **Avoid redundancy:** phrases like “reshape sectors” and “technological transformation” are used repeatedly.
3. **Ensure precision:** clarify ambiguous phrases such as “Copilot, ChatGPT, and Bard” are these examples of large language models, generative tools, or AI platforms?

Some example of academic writing tone. Kindly review the draft to identify them and correct accordingly.

1.	“AI is increasingly penetrating all sectors of the global economy…”

“AI is increasingly transforming sectors of the global economy…”

2.	“finds itself at the forefront…”

“is positioned at the forefront…”

3.	“must equip learners with the competencies…”

“should equip learners with the requisite competencies…”

4.	“AI tools such as Copilot, ChatGPT, and Bard…”

“AI tools—including generative systems like Copilot, ChatGPT, and Bard…”

5.	“ethical misuse and regulatory gaps”

“potential misuse and regulatory shortcomings”

6.	“cannot be realized without strong ethical frameworks…”

“depends upon the establishment of robust ethical frameworks

7.	“surpass human control and comprehension”

“outpace human oversight and understanding”

8. The IPS moderation result is discussed at length, which is good—but consider softening the tone slightly to account for sample-specific limitations:

“While IPS was not statistically significant in this context, its theoretical relevance remains compelling, particularly where robust policy infrastructure exists.

9. “Artificial Intelligence is reshaping global economies and educational paradigms, challenging traditional approaches to teaching and learning.” see this Consider integrating transitional phrases: For example,

“In light of this transformation…” or “Against this backdrop…” to signal a shift in focus.

**Improve on the scope of the study was poorly articulated:** There is need to contextualize the scope as to clearly show were the study was carried out and why. Clarify the scope of your study earlier. Perhaps add: “This paper focuses on the ethical integration of AI within TVET systems in developing countries, with an emphasis on institutional policy and educator behavior.”

**Suggested Improvements**

Clarity and Flow. Consider breaking longer sentences into shorter, more digestible units. For example:

1. “Ethical principles guiding AI adoption offer a multifaceted foundation for understanding how educators engage with ethical guidelines in practice” could be revised to: “Ethical principles provide a multifaceted foundation. They help explain how educators engage with guidelines in day-to-day practice.”
2. Phrases like “must be translated into concrete, enforceable practices to influence real-world outcomes” could be made more concise: "must be operationalized through enforceable practices to impact educational outcomes."
3. Avoid repeating similar ideas in adjacent sentences (e.g., the notion of institutional mechanisms transforming ideals into practice is echoed multiple times).
4. Use transitions to better link paragraphs. Phrases like “Building on this foundation…” or “This leads to…” help orient the reader.
5. The use of acronyms (EPG, CAEG, PAAT) is fine for brevity, but it may help to occasionally restate the full terms for readability—especially at the start of each hypothesis.

**Strengthen Empirical Gap.** The final paragraph acknowledges a lack of empirical studies in TVET settings, especially in developing countries. You could amplify this by highlighting why Nigeria presents a particularly compelling case (e.g., rapid digital expansion, policy vacuums, educator training gaps).

Clarify Theoretical Framing; You may want to clarify whether your proposed model is grounded in a specific theory (e.g., institutional theory, stakeholder theory, or ethics of care) to strengthen academic framing.

Methodology section

Clear Research Design, A cross-sectional survey design is aptly justified and well-aligned with the objectives of examining relationships among latent constructs. Contextual Relevance, You situate the study effectively within Nigeria’s emerging AI-in-education ecosystem, highlighting local institutional challenges. Instrument Robustness, Multiple measures (face/content validity, expert review, pilot testing) strengthen your instrument’s credibility. Using validated scales from recent literature boosts construct validity. Sampling Transparency, The demographics are clearly presented, with informative stats on gender and qualification levels. PLS-SEM Rationale and Execution

Discussion Section

**Area of strength**

1. Insightful Findings Interpretation: The triangulation of direct, mediating, and moderating effects is well-executed. You offer layered insight into how ethical principles translate into practice and shape perceptions of AI adoption.

2. Evidence-Based Argumentation: Your extensive engagement with relevant literature reinforces theoretical alignment and empirical relevance.

3. Real-World Relevance: The connection to Nigeria’s TVET ecosystem and resource constraints provides a compelling backdrop for the findings and implications.

4. Theoretical Contribution: Introducing Rest’s Four-Component Model adds originality. It also grounds your results in a moral psychology framework—a fresh angle in AI ethics literature.

5. Action-Oriented Implications: The dual strategy (bottom-up ethical awareness and top-down policy reforms) offers clear, feasible policy recommendations for institutions in developing countries.

**Clear separate this part to enhance readability and flow of thought**

1. Interpretation of Findings

2. Theoretical Implications

3. Policy Implications

4. Practical Implications

Avoid starting sentences with “Ultimately” or “While…” too often. They work well occasionally, but varying sentence openers improves rhythm.

Example tweak: “Ultimately, our study confirms…” “This study confirms…”

You might vary tone by swapping formal phrasing occasionally: “Our model provided empirical support…” “The findings offer empirical support…”

3. Expand Practical Implications Briefly: Consider adding one line on how curriculum developers or educators might act on these insights—for example, integrating AI ethics into training modules.
