# Supplementary material for: Ethical compliance and institutional policy support for artificial intelligence integration in African TVET Education: A structural equation modeling approach
Source: PLoS One. 2025 Dec 19;20(12):e0335747. doi: 10.1371/journal.pone.0335747 (PMC12716723; doi:10.1371/journal.pone.0335747)
Supplement: Attachment — Submitted filename: Response to Reviewers Comments.docx [file pone.0335747.s003.docx]

September 2, 2025

Dear Editor-in-chief of Plos One

We would like to thank the reviewers for their insightful comments that have helped us to improve our manuscript and we thank the Editor for the opportunity to revise the manuscript title “Ethical Compliance and Institutional Policy Support for Artificial Intelligence Integration in African TVET Education: A Structural Equation Modeling Approach”. We revised the manuscript considering all the comments from the reviewers. *W*e inked our inputs red across the sections concerned. See the details below:

**Responses to Reviewers’ Comments**

| Reviewer Comment | Author Response |
| --- | --- |
| Reviewer #1 |  |
| Break up long paragraphs for better readability and academic flow. | We appreciate the suggestion. We have revised the introduction by breaking up long paragraphs into shorter, digestible segments to enhance academic flow and readability. |
| Avoid redundancy: phrases like “reshape sectors” and “technological transformation” are used repeatedly. | Thank you. We have carefully reviewed the manuscript and eliminated redundant expressions to improve precision and stylistic variety. |
| Clarify ambiguous terms like “Copilot, ChatGPT, and Bard.” | We have clarified that these are generative AI tools and revised the phrase to read: “AI tools, including generative systems like Copilot, ChatGPT, and Bard…” |
| Revise academic tone (e.g., "must equip" → "should equip") | We have revised informal or overly assertive phrases throughout the manuscript to align with appropriate academic tone, as exemplified in the provided suggestions. |
| Soften tone around IPS moderation result. | We have adjusted the tone of our discussion on IPS moderation. The sentence now reads: “While IPS was not statistically significant in this context, its theoretical relevance remains compelling…” |
| Improve transitions and academic rhythm. | Transitional phrases like “In light of this transformation…” and “Building on this foundation…” have been added to improve coherence between paragraphs. |
| Clarify the scope of the study early. | We have clarified the study’s scope in the introduction: “This paper focuses on the ethical integration of AI within TVET systems in developing countries, with an emphasis on institutional policy and educator behavior.” |
| Shorten and split complex sentences. | Several complex sentences were split or simplified to improve clarity and ease of comprehension, including those referring to operationalization of ethical principles. |
| Avoid redundancy across adjacent sentences. | We reviewed the manuscript for repetitive content and have eliminated or consolidated ideas that were echoed multiple times. |
| Restate full terms of acronyms (e.g., EPG) at key points. | We have reintroduced full terms at the beginning of each hypothesis and major section for clarity. |
| Strengthen justification for Nigeria as context. | We have elaborated on why Nigeria offers a compelling case for the study—highlighting digital expansion, AI policy gaps, and limited ethical AI training. |
| Clarify theoretical framing in literature review. | We have expanded our explanation of Rest’s Four-Component Model and its alignment with each construct in the conceptual framework. |
| Reviewer #2 |  |
| Keywords can be improved. | We revised the keywords to include “African education,” “digital education,” and “AI in education” to better reflect the thematic scope. |
| Compare with recommended literature to sharpen research gap. | We reviewed the suggested literature and incorporated comparisons to show our unique contribution—particularly the focus on moral psychology in AI adoption among TVET educators. |
| Revise RQ3 and RQ4 to show how influence occurs. | We revised the research questions to emphasize causal mechanisms, e.g., “How does institutional policy support influence the relationship between…” |
| Shorten the introduction; delete unnecessary parts. | We have significantly reduced the length of the introduction, focusing only on critical contextual and conceptual background. |
| Move theoretical basis explanation to literature review. | We moved the detailed discussion of Rest’s moral psychology model into the literature review to align with standard academic structure. |
| Each variable should be explained in the literature review. | We provided an expanded explanation of each variable in the literature review, supplemented by a copyable summary table. |
| Redraw the model diagram for academic professionalism. | We have redesigned the conceptual model using academic illustration tools (e.g., SmartPLS, Lucidchart) to enhance visual clarity and scholarly quality. |
| Add post-results model diagram with path coefficients. | A verified model diagram showing path coefficients and significance levels has been added after the results section. |
| Separate discussion section clearly by headings. | The discussion section is now divided into: Interpretation of Findings, Theoretical Implications, Policy Implications, and Practical Implications. |
| Avoid repetitive sentence openers like “Ultimately” or “While…” | We varied our sentence openers and replaced repetitive ones to improve narrative flow and academic rhythm. |
| Expand practical implications. | We added specific examples for curriculum developers and educators—e.g., incorporating AI ethics modules in teacher training programs. |

Sincerely,

On behalf of all co-authors.
